# Comparative studies on the intestinal health of wild and cultured ricefield eel (*Monopterus albus*)

**DOI:** 10.3389/fimmu.2024.1411544

**Published:** 2024-06-10

**Authors:** Hang Yang, Quan Yuan, Mohammad Mizanur Rahman, Weiwei Lv, Weiwei Huang, Wei Hu, Wenzong Zhou

**Affiliations:** ^1^ Eco-environmental Protection Research Institute, Shanghai Academy of Agricultural Sciences, Shanghai, China; ^2^ Key Laboratory of Integrated Rice-Fish Farming Ecosystem, Ministry of Agriculture and Rural Affairs, Shanghai Academy of Agricultural Sciences, Shanghai, China; ^3^ Institute of Marine Sciences, University of Chittagong, Chattogram, Bangladesh; ^4^ State Key Laboratory of Freshwater Ecology and Biotechnology, Institute of Hydrobiology, Chinese Academy of Sciences, Hubei Hongshan Laboratory, Wuhan, China

**Keywords:** *Monopterus albus*, intestinal health, gut barrier, intestinal microbiota, intestinal tissue morphology

## Abstract

Fish intestinal health under intensive aquaculture mode plays an important role in growth, development, and immune function. The present study was aimed to systematically investigate the differences of intestinal health between wild and cultured *Monopterus albus* by biochemical parameters, histomorphology, and molecular biology. A total of 15 healthy *M. albus* per group, with an average body weight of 45 g, were sampled to analyze intestinal health parameters. Compared with wild fish, the cultured *M. albus* in the foregut had lower trypsin, lipase, SOD, CAT, T-AOC, and GSH-Px activities (*P* < 0.05) and higher amylase activity and MDA content (*P* < 0.05). The villus circumference and goblet cells in the cultured group were significantly lower than those in the wild group (*P* < 0.05). In addition, the cultured fish showed lower relative expression levels of *occludin*, *zo-1*, *zo-2*, *claudin-12*, *claudin-15*, *mucin5*, *mucin15*, *lysozyme*, *complement 3*, *il-10*, *tgf-β1*, *tgf-β2*, and *tgf-β3* (*P* < 0.05) and higher *il-1β*, *il-6*, *il-8*, *tnf-a*, and *ifnγ* mRNA expressions than those of wild fish (*P* < 0.05). In terms of gut microbiota, the cultured group at the phylum level displayed higher percentages of *Chlamydiae* and *Spirochaetes* and lower percentages of *Firmicutes*, *Bacteroidetes*, *Actinobacteria*, *Cyanobacteria*, and *Verrucomicrobia* compared to the wild group (*P* < 0.05). At the genus level, higher abundances of *Pseudomonadaceae_Pseudomonas* and *Spironema* and lower abundances of *Lactococcus* and *Cetobacterium* were observed in the cultured group than in the wild group (*P* < 0.05). To our knowledge, this is the first investigation of the intestinal health status between wild and cultured *M. albus* in terms of biochemistry, histology, and molecular biology levels. Overall, the present study showed significant differences in intestinal health between wild and cultured *M. albus* and the main manifestations that wild *M. albus* had higher intestinal digestion, antioxidant capacity, and intestinal barrier functions than cultured *M. albus.* These results would provide theoretical basis for the subsequent upgrading of healthy aquaculture technology and nutrient regulation of intestinal health of cultured *M. albus.*

## 1 Introduction

The global demand for fish has been accelerating owing to the increased population and raised awareness of the health benefits of fish consumption instead of meat products. The main sources of fish are natural resources (i.e., artisanal fisheries) and aquaculture (1). However, the increase in total fish consumption and unsustainable fishing operations have made it impossible to meet the growing demand with wild-caught fish alone (2), and the aquaculture industry has become a suitable means to satisfy the global demand for fish supply (3). In recent years, the wide acceptance of artificial compound feeds coupled with the continuous optimization of nutritional balancing has led to an increase in farmed fish production year by year (4). However, with the higher demand for unit production and the frequent occurrence of fish diseases, researchers are also conceding the vital relationship between growth performances and immune function in accord to intestinal health, a key indicator organ of fish health.

Fish intestinal health is a complex and comprehensive assessment system when considering the important role of the intestine in the organism, mainly involving the digestive and antioxidant enzymes, tissue morphology, various barrier functions, and microorganism composition (5), which covers a wide range of physiological functions. Factors that cause differences in intestinal health between wild and cultured fish include the living environment (water temperature, water quality, and water salinity), developmental stage, and dietary sources (6). Compared to cultured fish, wild fish live in environments with a high level of dissolved oxygen, low living density, and low ammonia–nitrogen level, but excess heavy metal contents in the wild environment may also be detrimental to intestinal health (7). Wild fish have a richer diet composition, and the food they consume may have specific nutrients that are easily digested to influence intestinal health, although a lower intake as well as excessive consumption can also lead to slower growth rates (8).

The Asian swamp eel *Monopterus albus*, known as rice field eel, an important traditional aquaculture species in China with a culture production of 334,000 tons in 2022 (9), is preferred due to its tasty flesh and high nutritional and medicinal value. However, currently, few studies have been reported on the intestinal health of wild and cultured fish of *Paralichthys adspersus* (10), *Seriola lalandi* (11), *Huso dauricus* (12), *Oreochromis niloticus* (13), and *Genypterus chilensis* (14), which mainly focused on the intestinal microorganism composition, while other aspects were poorly investigated. However, health status is a comprehensive characteristic system, which is difficult to be evaluated by only a few parameters. Therefore, this study aimed to analyze the relationship among the digestive enzymes, antioxidant enzymes, intestinal barrier function, and microbial composition of *M. albus* from biochemistry, histology, and molecular biology levels in order to systematically compare the intestinal health of farmed and wild *M. albus*. The results would provide a theoretical basis for the subsequent upgrading of healthy aquaculture technology and nutrient regulation of intestinal health of cultured *M. albus.*

## 2 Materials and methods

The Animal Ethics Committee of Shanghai Academy of Agricultural Sciences approved all animal procedures.

### 2.1 Sample collection

In this work, healthy wild (*n* = 15) and cultured (*n* = 15) *M. albus* were collected, with an average body weight of 45.3 ± 5.1 and 44.2 ± 3.8 g, and obtained from Jinshan District, Shanghai (30.78° N, 121.18° E) and Zhuanghang Comprehensive Experiment Station of Shanghai Academy of Agricultural Sciences (30.89°, 121.41° E), respectively. Among them, cultured *M. albus* with an initial body weight of 15.2 ± 0.5 g were fed commercial diet containing 43% crude protein and 7% crude lipid at 16:00 each day by hand for 10 weeks, and the daily feeding rate was 3% to 5% of the body weight. During the feeding trial, the water was continuously aerated, and one-third of the aeration water was replaced daily (dissolved O_2_≥ 5.8 mg/L, water temperature 28 ± 2°C, pH 7.3 ± 0.2, and NH^4+^–N < 0.5 mg/L).

Before sampling, the *M. albus* fish were fasted for at least 48 h to ensure that no chyme was observed in the intestines. The fish were dissected under aseptic conditions, and the foregut and hindgut were removed with a sterilized scalpel. A portion of the foregut was taken and stored frozen at -20°C for intestinal digestive enzyme and antioxidant parameter analysis, another portion of the foregut was placed in liquid nitrogen for intestinal gene expression determination, and the other portion of the foregut was collected and fixed in Bouin’s solution for observation of tissue morphology. The hindgut per fish was put into RNase-free tubes and stored in liquid nitrogen for intestinal microbiota analysis. Three fish were pooled as one sample, with a total of 15 fish per group (each with five replicates), to perform analyses of the digestive enzymes, antioxidant parameters, and intestinal microbiota as well as real-time quantitative PCR.

### 2.2 Sample analysis

#### 2.2.1 Analysis of intestinal digestive enzymes and antioxidant parameters

The foregut samples were weighed and added four times the volume of pre-cooled saline. After homogenization, the samples were centrifuged at 4°C for 10 min (6,000 r/min), and the supernatant was extracted and stored at 4°C for the determination of intestinal digestive enzymes and antioxidant parameters within 24 h. The superoxide dismutase (SOD), glutathione peroxidase (GSH-Px), catalase (CAT), and total antioxidant capacity (TAOC) activities and malondialdehyde (MDA) content in the foregut were measured by using the corresponding kits produced by Nanjing Jiancheng Bioengineering Institute (Nanjing, China).

#### 2.2.2 Intestinal tissue morphology

The foregut samples were fixed in Bouin’s solution for at least 48 h and then dehydrated with ethanol, transparent with xylene, embedded in paraffin, sectioned (8 μm), and stained with hematoxylin–eosin (H&E). The sections were photographed under an optical microscope to observe the intestinal morphology parameters (Nikon YS100 micrographic system). The goblet cell amounts were determined according to Shi et al. (15).

#### 2.2.3 Real-time quantitative PCR analysis

Total RNA from intestine samples was extracted by using Trizol reagent following the manufacturer’s protocol, and the concentration and the purity of RNA were detected by using a UV spectrophotometer and agarose gel electrophoresis, respectively. Subsequently, cDNA synthesis was performed using the PrimeScript™ RT reagent kit (Takara, Dalian, China) and was then stored at –80°C until use. All real-time quantitative PCR analyses were performed using the SYBR^®^ Premix Ex Taq (Perfect Real-Time) kit (TaKaRa) according to the manufacturer’s instructions. The total reaction volume was 20 μL, containing 10 μL SYBR^®^ Premix Ex Taq™ (Tli RNaseH Plus), 0.5 μL upstream primer, 0.5 μL downstream primer, 1 μL cDNA template, and 8 μL ddH_2_O. The reaction program of real-time quantitative PCR was as follows: pre-denaturation at 95°C for 30 s, 35 cycles of denaturation at 95°C for 5 s, annealing at 58°C for 15 s, and extension at 72°C for 20 s; finally, the melting curve was performed to confirm the specificity. The PCR primers were obtained and designed based on *M. albus* sequences in the GenBank accession (**Table 1**), and RPL-17 was selected as a reference gene. The relative expression levels in intestine tissues were calculated by using the 2^-ΔΔCt^ method.

**Table 1:** Primer sequence of q-PCR.

Gene	Primer sequence (5′–3′)	Accession no.
*rpl-17*	F-AGAAATGCCCCATCTCCA	XM_020587712.1
	R-CCCTGTCTCCGTCTTGTTG	
*occludin*	F-TGTCGGGGAGTGGGTAAA	XM_020599328.1
	R-TCCAGGCAAATAAAGAGGCT	
*zo-1*	F-GGCATCATCCCCAACAAA	XM_020621576.1
	R-GCGAAGACCACGGAACCT	
*Zo-2*	F-AGCCGAGGTCGCACTTTA	XM_020615114.1
	R-GCTTTGCTTCTGTGGTTGAT	
*claudin-12*	F-TCACCTTCAATCGCAACG	XM_020607277.1
	R-ATGTCTGGCTCAGGCTTATCT	
*claudin-15*	F-CTCGCTGCTTGCTTTGACT	XM_020611334.1
	R-TTGAAGGCGTACCAGGACA	
*il-6*	F-TGAGTGCCGACCCAGTTT	XM_020606850.1
	R-CTTCAACCAGCCTATGGAGAC	
*il-8*	F-TACTGGTTCTGCTTACTGTCGC	XM_020597077.1
	R-CAAATCTTTTGCCCATCCCT	
*il-10*	F-TTTGCCTGCCAAGTTATGAG	XM_020593225.1
	R-CATTTGGTGACATCGCTCTT	
*il-1β*	F-GAGATGTGGAGCCCAAACTT	KM113037.1
	R-CTGCCTCTGACCTTCTGGACTT	
*tnf-a*	F-TTTCAAGGAGGGCTGGTTCT	XM_020624826.1
	R-CTTGACCAGCGCATCACTGT	
*Ifnγ*	F-GTCTGTCTGTCCCTCTGGCTAT	NM_001360732.1
	R-TTGGGGTGGGCAGATTTT	
*tgf-β1*	F-AACCCACTACCTCACTACCCG	XM_020605575.1
	R-GCCGAAGTTGGAAACCCT	
*tgf-β2*	F-ATTACGCCAAGGAGGTGC	XM_020622328.1
	R-GGGTTTTGAAGACGGAAGAT	
*tgf-β3*	F-AGTTTGTCGCTATCCACTTGC	XM_020590885.1
	R-GATGAGTTCCTTGGTGCTGTTA	
*mucin 5*	F-CAAGTCAGTTGCCAAAATCC	XR_002276876.1
	R-CCAAGCAGCTCAGGGTCT	
*mucin 15*	F-AGAAATGCCCCATCTCCA	XM_020608782.1
	R-CCCTGTCTCCGTCTTGTTG	
*lysozyme*	F-GGGAGAAATAAAGGTGAGGATG	XM_020600993.1
	R-CAGATGAGTTGACAAGGCAGTT	
*complement 3*	F-TTGATGTTCCCCTGCGTTAT	XM_020588963.1
	R-CACCTGCTCTACCTGCTTGTC	

#### 2.2.4 Intestinal microbiota analysis

DNA extraction and PCR amplification were performed according to the instructions of the E.Z.N.A.^®^ soil kit (Omega Bio-tek, Norcross, GA, USA), DNA concentration and purity were examined using NanoDrop2000, and the quality of the DNA extractions was examined using 1% agarose gel electrophoresis. The quality of the DNA extractions was determined using 338F (5′-ACTCCTACGGGGAGGCAGCAG-3′) and 806R (5′-GGACTACHVGGGTWT CTAAT-3′) primers for PCR amplification of the V3–V4 variable region. The PCR product was extracted from 2% agarose gel and purified using PCR Clean-Up Kit (YuHua, Shanghai, China) according to the manufacturer’s instructions and quantified using QuantiFluor™-ST (Promega, USA). Bioinformatic analysis was carried out using Illumina’s NovaSeq 6000 platform by Shanghai Personal Biotechnology Co., Ltd. The detailed information of intestinal microorganisms such as α-diversity, β-diversity, composition, and abundance were analyzed by the online platform of Personal Cloud Platform (www.genescloud.cn). The raw data are deposited in the National Center for Biotechnology Information (NCBI) sequence read archive (SRP504613).

### 2.3 Statistical analysis

The experimental results were expressed as mean with standard deviation. All data were subjected to equality of variances with Levene’s test with SPSS 22.0 software, and independent-sample *t*-tests were used to determine significant differences at *P* < 0.05.

## 3 Results

### 3.1 Intestinal digestive enzymes

As shown in **Table 2**, wild *M. albus* had higher activities of trypsin and lipase (*P* < 0.05) but lower activities of amylase than cultured *M. albus* (*P* < 0.05). Meanwhile, extremely significant differences were noted for lipase and amylase activities (*P* < 0.01).

**Table 2:** Intestinal digestive enzymes of the wild and cultured *M. albus*.

Index	Wild	Cultured	*P*-value
Trypsin (U/mg prot)	947.4 ± 39.2	876.2 ± 41.1	0.023*
Lipase (U/g prot)	35.98 ± 3.71	26.69 ± 1.39	0.001**
Amylase (U/mg prot)	0.73 ± 0.05	1.16 ± 0.12	<0.001**

Values marked with asterisks are significantly different (*P < 0.05 and **P < 0.01).

### 3.2 Intestinal antioxidant parameters

As shown in **Table 3**, the intestinal antioxidant enzymes of the wild and cultured *M. albus* demonstrate very significant differences in this study (*P* < 0.01). Compared with the cultured group, increased SOD, CAT, GSH-Px, and T-AOC activities and a decreased MDA level were found in the wild *M. albus* group (*P* < 0.01).

**Table 3:** Intestinal antioxidant enzymes of the wild and cultured *M. albus*.

Index	Wild	Cultured	*P*-value
SOD (U/mg prot)	985.2 ± 41.2	790.7 ± 97.3	0.003**
CAT (U/mg prot)	8.14 ± 0.37	3.11 ± 0.52	<0.001**
GSH-Px (U/mg prot)	174.2 ± 9.8	115.0 ± 11.3	<0.001**
T-AOC (mmol/g prot)	130.6 ± 9.7	86.00 ± 8.55	<0.001**
MDA (nmol/mg prot)	2.05 ± 0.21	3.40 ± 0.50	0.001**

Values marked with asterisks are significantly different (*P < 0.05 and **P < 0.01).

SOD, superoxide dismutase; CAT, catalase; GSH-Px, glutathione peroxidase; T-AOC, total antioxidant capacity; MDA, malondialdehyde.

### 3.3 Intestinal tissue morphology

**Figure 1:**
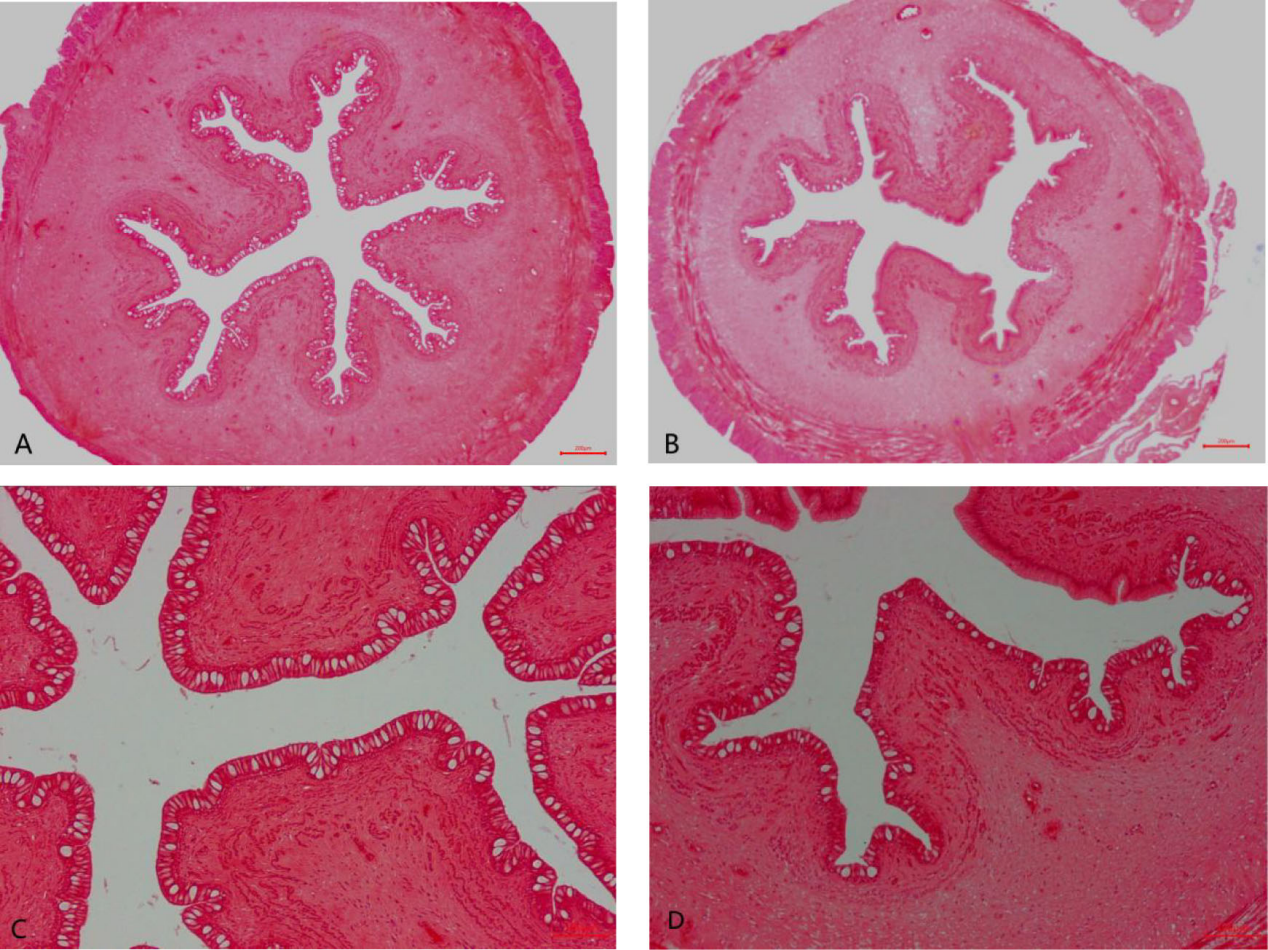
Foregut histological structure of the wild and cultured *M. albus* (H&E staining). **(A)** Wild *M. albus* (×40), **(B)** cultured *M. albus* (×40), **(C)** wild *M. albus* (×100), and **(D)** cultured *M. albus* (×100).

The intestinal histology of the foregut is displayed in **Table 4** and **Figure 1**. Significant changes were observed for villus circumference (VC) and goblet cell amount (GCA) (*P* < 0.05). The values of VC and GCA in wild *M. albus* presented to be higher than those in cultured *M. albus* (*P* < 0.01).

**Table 4:** Intestinal morphology of the wild and cultured *M. albus*.

Index	Wild	Cultured	*P*-value
Villus circumferences (μm)	10,701.76 ± 859.7	8,061.34 ± 505.1	<0.001**
Goblet cell amounts (A/root)	200.6 ± 15.3	120.2 ± 11.2	<0.001**

Values marked with asterisks are significantly different (*P < 0.05 and **P < 0.01).

### 3.4 Intestinal chemical barrier

**Figure 2:**
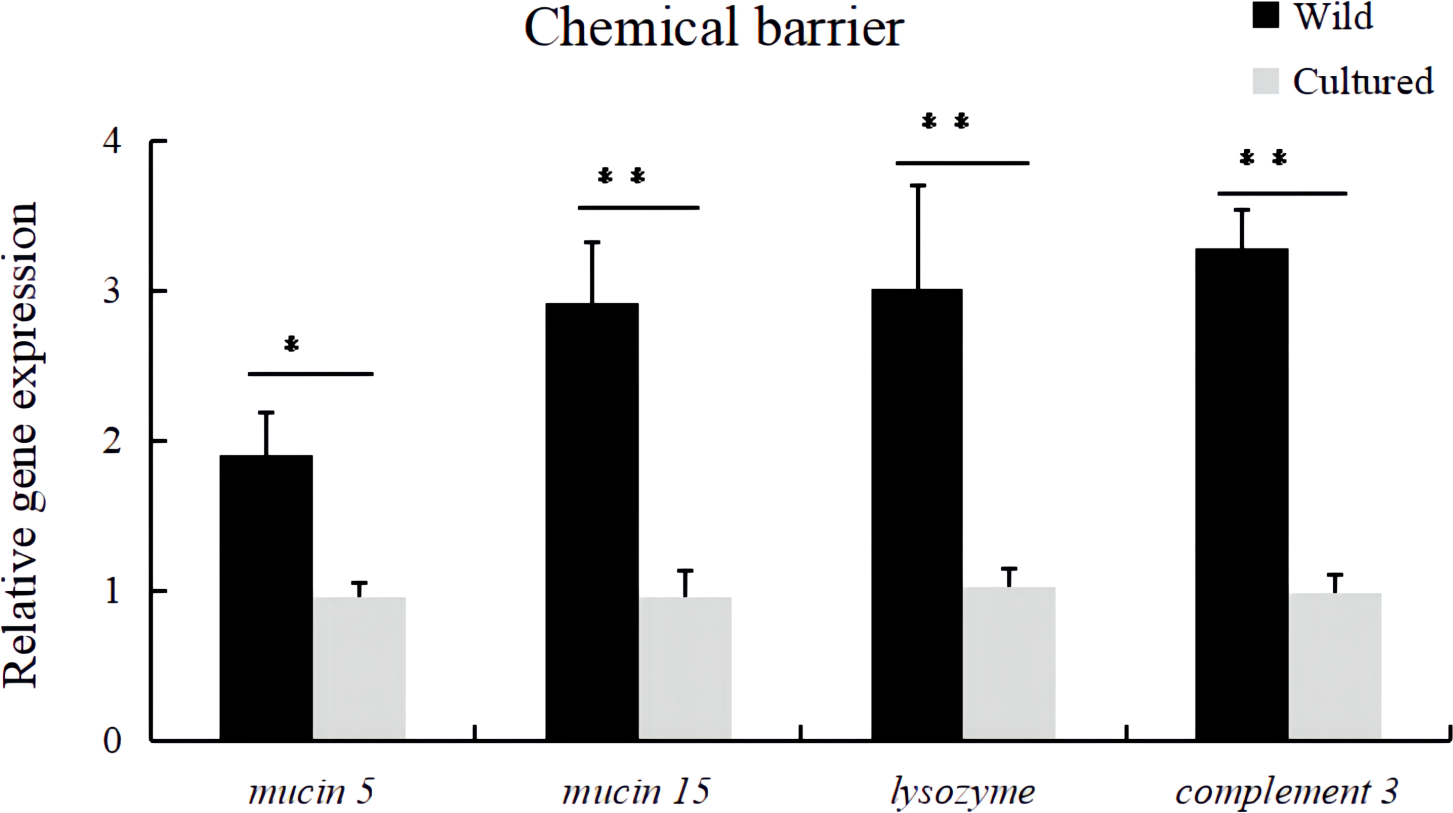
Relative expression levels of chemical barrier-related genes (*mucin 5*, *mucin 15*, *lysozyme*, and *complement 3*) in the intestine of the wild and cultured *M. albus*. Values marked with asterisks are significantly different (*P < 0.05 and **P < 0.01).

Compared with the wild fish, the relative expression levels of *mucin5*, *mucin15*, *lysozyme*, and *complement 3* mRNA in the intestine were downregulated in cultured *M. albus* (**Figure 2**) (*P* < 0.05).

### 3.5 Intestinal physical barrier

**Figure 3:**
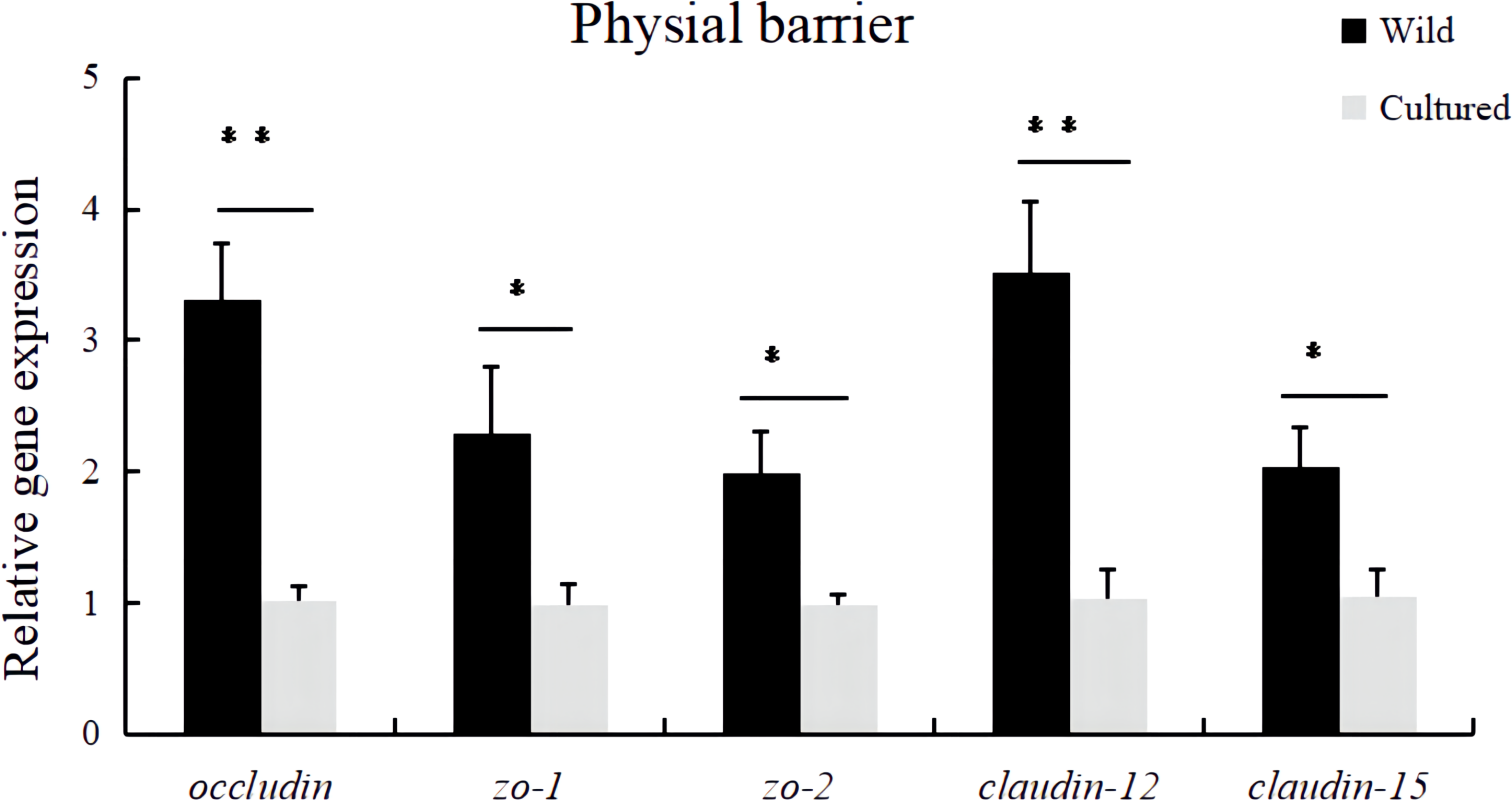
Relative expression levels of physical barrier-related gene (*occludin*, *zo-1*, *zo-2*, *claudin-12*, and *claudin-15*) in the intestine of the wild and cultured *M. albus*. Values marked with asterisks are significantly different (*P < 0.05 and **P < 0.01).

In **Figure 3**, the *occludin*, *zo-1*, *zo-2*, *claudin-12*, and *claudin-15* mRNA expression levels in the intestine of cultured *M. albus* were significantly lower than those of wild *M. albus* (*P* < 0.05).

### 3.6 Intestinal immunological barrier

**Figure 4:**
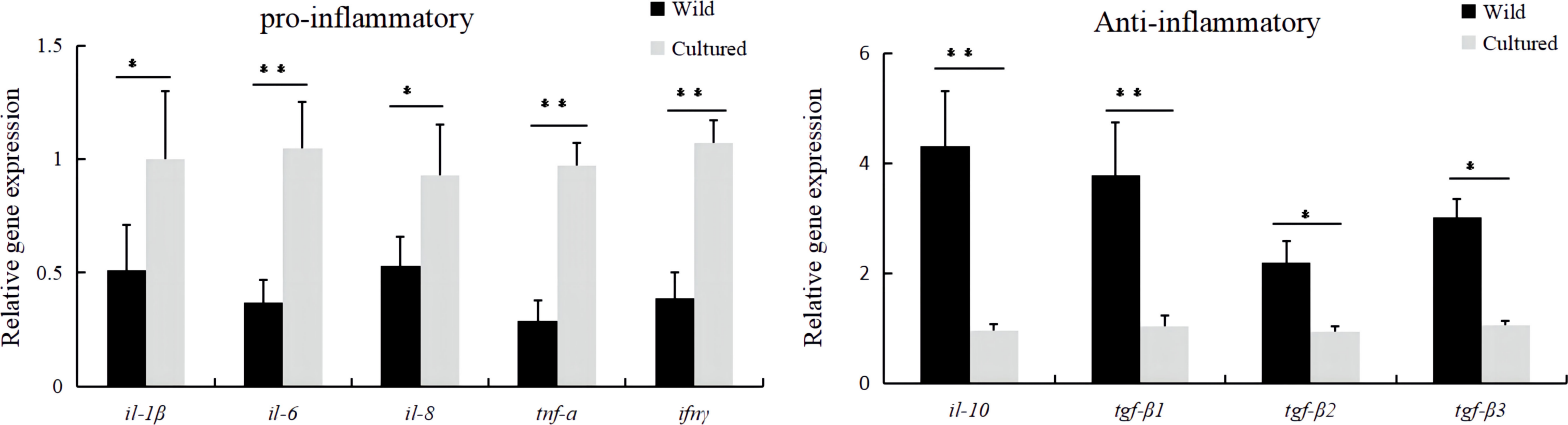
The mRNA expressions of inflammatory-related genes in the intestine of the wild and cultured *M. albus*. Values marked with asterisks are significantly different (*P < 0.05 and **P < 0.01).

In terms of inflammation-related genes, the mRNA expressions of pro-inflammatory genes (*il-1β*, *il-6*, *il-8*, *tnf-a*, and *ifnγ*) and anti-inflammatory genes (*il-10*, *tgf-β1*, *tgf-β2*, and *tgf-β3*) were significantly affected (*P* < 0.05). The cultured group had significantly higher relative mRNA levels of *il-1β*, *il-6*, *il-8*, *tnf-a*, and *ifnγ* and lower relative mRNA levels of *il-10*, *tgf-β1*, *tgf-β2*, and *tgf-β3* in the intestine of *M. albus* compared to the wild group (**Figure 4**).

### 3.7 Intestine microbiota analysis (microbiological barrier)

In order to have a more comprehensive assessment of the alpha diversity of the gut microbial communities of wild and cultured *M. albus*, this study characterized richness by Chao1 and observed species, diversity by Shannon and Simpson, and coverage by Goods coverage.

As can be seen from **Table 5**, a significant difference was observed in the α-diversity of the intestinal microbiota of cultured and wild groups. The coverage index is close to 1, which indicates that the bacterial community was adequately sampled and the data are representative of the population. Chao1, observed species, and Shannon and Simpson indices were found to be significantly higher in the wild than cultured *M. albus*, implying that wild *M. albus* had higher richness and diversity of intestinal flora.

**Table 5:** α-diversity of intestinal microbiota of the wild and cultured *M. albus*.

Index	Wild	Cultured	*P*-value
Chao1	559.25 ± 53.24	302.8 ± 88.13	0.005**
Observed species	550.73 ± 49.13	294.90 ± 85.64	0.004**
Shannon	6.72 ± 0.38	3.48 ± 0.98	<0.001**
Simpson	0.95 ± 0.05	0.74 ± 0.12	0.017*
Goods coverage	1.00 ± 0.00	1.00 ± 0.00	0.798

Values marked with asterisks are significantly different (*P < 0.05 and **P < 0.01).

**Figure 5:**
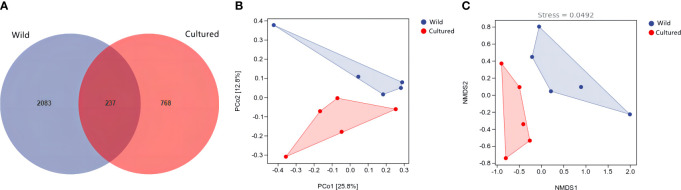
Venn diagram of operating taxonomic units **(A)** and β-diversity analysis **(B, C)** for the intestine microbiota of the wild and cultured *M. albus*.

The Venn diagram of operating taxonomic units (OTUs) is shown in **Figure 5A**, with 237 shared OTUs and 2,083 and 768 unique OTUs in the wild and cultured groups, respectively. Meanwhile, the β-diversity was displayed by principal coordinate analysis (PCoA) and non-metric multidimensional scaling analysis (NMDS). As shown in **Figures 5B, C**, there were no significant overlaps between the wild and cultured groups in PCoA (*P* = 0.025) and NMDS (*P* = 0.014).

**Figure 6:**
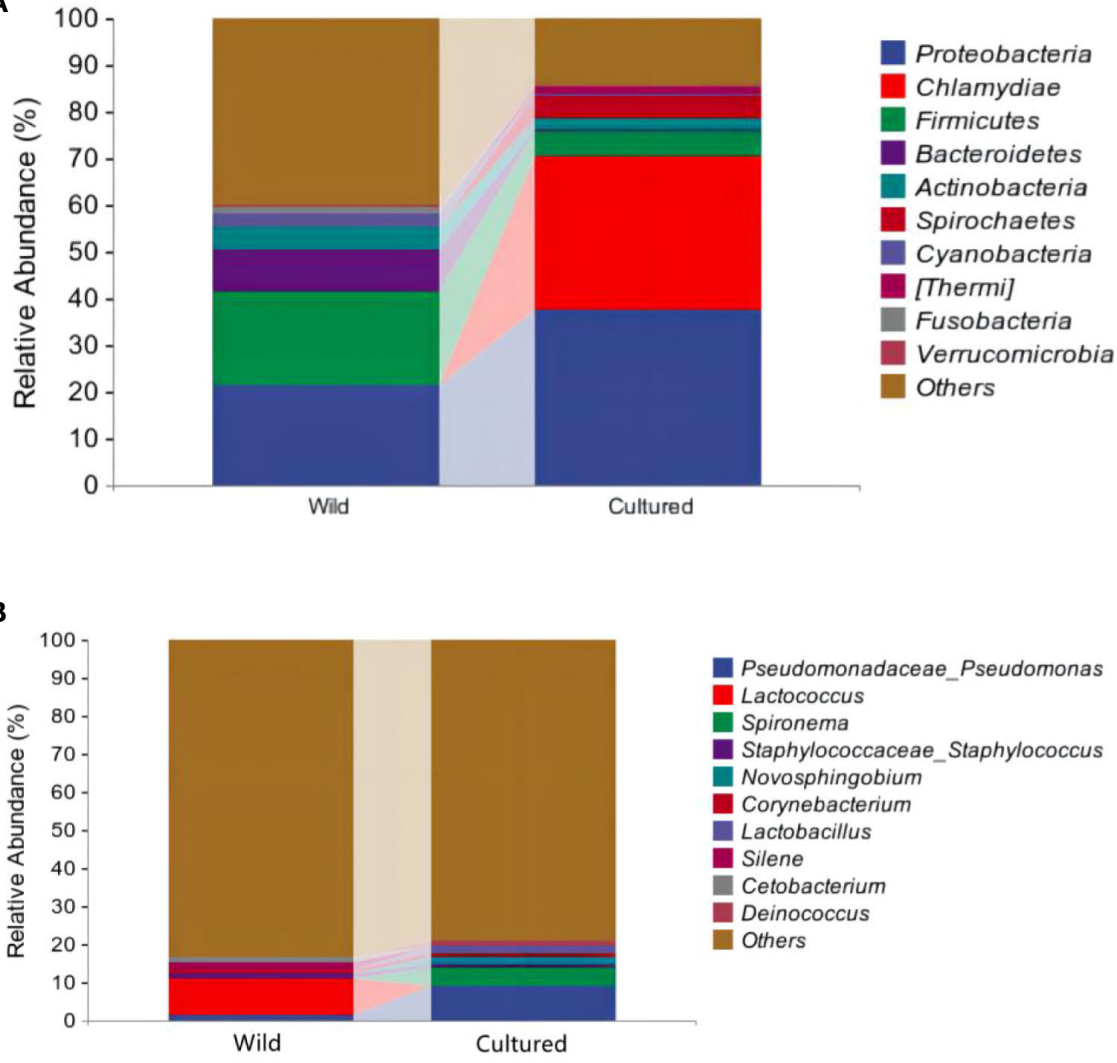
Community analysis at the phylum **(A)** and genus **(B)** levels for the intestinal microbiota of the wild and cultured *M. albus*.

As shown in **Figure 6A**, **Table 6**, the top 10 phyla according to the abundance of gut microbiota were observed as *Proteobacteria*, *Chlamydiae*, *Firmicutes*, *Bacteroidetes*, *Actinobacteria*, *Spirochaetes*, *Cyanobacteria*, *Thermi*, *Fusobacteria*, and *Verrucomicrobia*. The dominant phyla in the wild group were *Proteobacteria* (21.26%) and *Firmicutes* (20.50%) and in the cultured group were *Proteobacteria* (37.66%) and *Chlamydiae* (32.73%). Compared to the wild group, the cultured group had a higher abundance of *Chlamydiae* and *Spirochaetes* and a lower abundance of *Firmicutes*, *Bacteroidetes*, *Actinobacteria*, *Cyanobacteria*, and *Verrucomicrobia*.

**Table 6:** Dominant bacteria and relative abundance (%) at phylum and genus levels for the intestinal microbiota of the wild and cultured *M. albus*.

Index	Wild	Cultured	*P*-value
Phylum level			
*Proteobacteria*	21.26 ± 15.06	37.66 ± 27.53	0.184
*Chlamydiae*	0.04 ± 0.03	32.73 ± 9.39	0.001**
*Firmicutes*	20.50 ± 14.58	5.03 ± 2.24	0.047*
*Bacteroidetes*	8.95 ± 4.63	0.84 ± 0.73	0.022*
*Actinobacteria*	5.87 ± 2.83	2.63 ± 0.96	0.041*
*Spirochaetes*	0.02 ± 0.01	4.75 ± 2.09	0.003**
*Cyanobacteria*	3.25 ± 1.41	0.44 ± 0.51	0.016*
[*Thermi*]	1.11 ± 0.10	2.83 ± 1.35	0.130
*Fusobacteria*	1.71 ± 1.03	1.07 ± 0.05	0.245
*Verrucomicrobia*	0.66 ± 0.26	0.11 ± 0.12	0.019*
Others	36.84 ± 22.37	12.48 ± 5.7	0.111
Genus level			
*Pseudomonadaceae_Pseudomonas*	1.35 ± 0.8	9.01 ± 4.92	0.020*
*Lactococcus*	9.42 ± 2.93	0.00 ± 0.00	0.017*
*Spironema*	0.00 ± 0.00	4.75 ± 1.21	0.025*
*Staphylococcaceae_Staphylococcus*	1.72 ± 0.05	1.20 ± 0.19	0.124
*Novosphingobium*	1.00 ± 0.50	1.89 ± 0.95	0.065
*Corynebacterium*	0.88 ± 0.10	0.83 ± 2.29	0.301
*Lactobacillus*	1.03 ± 0.21	1.67 ± 0.89	0.214
*Silene*	0.69 ± 0.39	1.00 ± 0.50	0.117
*Cetobacterium*	1.56 ± 0.95	0.02 ± 0.01	0.008**
*Deinococcus*	1.05 ± 0.11	1.53 ± 0.83	0.069
Others	81.3 ± 20.16	79.11 ± 17.4	0.913

Values marked with asterisks are significantly different (*P < 0.05 and **P < 0.01).

At the genus level (**Figure 6B**; **Table 6**), the top 10 intestinal microorganisms were recorded as *Pseudomonadaceae_Pseudomonas*, *Lactococcus*, *Spironema*, *Staphylococcaceae_Staphylococcus*, *Novosphingobium*, *Corynebacterium*, *Lactobacillus*, *Silene*, *Cetobacterium*, and *Deinococcus*. *Lactococcus* (9.42%) and *Pseudomonadaceae_Pseudomonas* (9.01%) were found to be the dominant bacteria in the wild and cultured *M. albus*, respectively. Higher percentages of *Pseudomonadaceae_Pseudomonas* and *Spironema* and lower percentages of *Lactococcus* and *Cetobacterium* were observed in the cultured group than in the wild group.

## 4 Discussion

It is well known that the intestinal tract is an important site for the digestion and absorption of nutrients, while intestinal trypsin, lipase, and amylase are the main indicators of digestive function. The present study showed that wild *M. albus* had higher activities of trypsin and lipase and lower amylase than those of cultured ones, which may be due to the differences in ingestion capacities of varied diets in the living environments. The commercial diet may be rich in wide forms of carbohydrates, and the long-term dietary habits forced the cultured *M. albus* to adapt to high-starch diets, resulting in higher intestinal amylase activity. In addition, chronic satiation maybe also leads to weakened intestinal digestion (16). In wild environment, *M. albus* mainly ingest fish and shrimp, aquatic earthworms, aquatic insects, and a few plant roots and algae, which is more in line with *M. albus*’s dietary habits, coupled with greater exercise in the wild situation. Therefore, the secretion and the activity of intestinal protease and lipase are higher in the wild group, which can be inferred from the intestinal tissue morphology as related to digestive and absorptive functions (17). The increased villus circumference could enhance digestion by promoting the contact area with feed, thus facilitating digestion. Goblet cells are scattered among columnar cells and are capable of secreting digestive fluid. The villus circumference and goblet cells amount in the wild group were higher than those in the cultured group (**Figure 1**), which ascertained the robust intestinal digestive and absorptive functions of wild *M. albus*.

In addition to the function of nutrient digestion and absorption, the intestine is also an important barrier for animals to resist external environmental stimuli. In normal metabolism, the dynamic balance of redox is always maintained, and when fish are subjected to internal and external coercion, a large number of reactive oxygen molecules are produced, and the balance of the antioxidant system and the repair system in the body is impaired, which will cause the destruction of the organism’s tissues and functions, ultimately leading to the reduction of intestinal health, immunity, and growth performance (18). SOD, CAT, GSH-Px, T-AOC, and MDA are important indicators of antioxidant function. SOD and CAT are able to scavenge free radicals to reduce the damage caused by oxidative stress to the intestinal mucosa, which plays an important role in intestinal defense and repair (19); GSH-Px mirrors the body’s ability to scavenge free radicals, which protects the animal from damage caused by lipid peroxides (20); T-AOC is a comprehensive indicator of antioxidant function, reflecting the ability to compensate for external stimuli and the state of free radical metabolism (19); and MDA content represents the degree of lipid peroxidation and indirectly the degree of cellular damage (21). The present study showed that the SOD, CAT, GSH-Px, and T-AOC activities in wild *M. albus* were significantly higher, while the MDA level was significantly lower compared with the cultured ones, indicating that the antioxidant capacity of wild *M. albus* is stronger than that of cultured *M. albus*.

Fish and terrestrial animals have similar intestinal barrier compositions, which mainly include biological barrier, chemical barrier, physical barrier, and immunological barrier (22), while barrier function plays an important role in maintaining intestinal homeostasis. When the intestinal barrier function of fish is damaged, it firstly affects the intestinal health, and then it is susceptible to the attack of pathogenic bacteria, which ultimately leads to a decline in growth performance (23).

Chemical barriers are chemicals such as mucins, complement proteins, lysozyme, and intestinal antimicrobial peptides in the outer mucus layer of intestinal epithelial cells (24). Currently, there are no documented comparisons so far on exploring the intestinal chemical barrier function in wild and cultured fish. In this study, significantly higher mRNA expressions of chemical barrier-related genes (*mucin 5*, *mucin 15*, *lysozyme*, and *complement 3*) have been observed in wild *M. albus* compared with cultured *M. albus*, implying the superior intestinal chemical barrier functionality of wild *M. albus*.

The intestinal physical barrier mainly consists of intestinal epithelial cells, which are connected to each other by adhesive junctions and tight junctions and considered to be an important determinant of intestinal cell permeability (25). The physical barrier can be strengthened by regulating tight junction proteins, which are a major determinant of the magnitude of intercellular permeability. The tight junctions are mainly composed of several membrane proteins, namely, occludin, claudins and adhesion factors, and zonula occludens (ZO) (26). However, the differences of intestinal tight junction proteins in wild and cultured fish have not yet been investigated. In this study, the wild group had a higher mRNA relative expression of *occludin*, *zo-1*, *zo-2*, *claudin-12*, and *claudin-15* in the gut than the cultured group, and these results provide a reference for future nutritional regulation in adjusting the integrity of the intestinal physical barrier in fish.

Inflammatory cytokines are important indicators of the intestinal immunological barrier in aquatic animals (27), which can usually be categorized into pro-inflammatory and anti-inflammatory cytokines based on the type of response effect (28). Pro-inflammatory cytokines play a role in inflammatory response by promoting inflammation; overexpression can exacerbate intestinal mucosal damage and can affect the distribution and expression of tight junction proteins, increasing intestinal permeability (29). Studies in fish have demonstrated that the downregulation of pro-inflammatory cytokine mRNA levels and upregulation of anti-inflammatory cytokine mRNA levels attenuate excessive inflammatory responses (30). Currently, no studies related to immunological barrier in wild and cultured *M. albus* have been reported. However, the present study revealed a reduced mRNA expression level of intestinal pro-inflammatory-related genes (*il-1β*, *il-6*, *il-8*, *tnf-a*, and *ifnγ*) and upregulated mRNA expression of anti-inflammatory genes (*il-10*, *tgf-β1*, *tgf-β2*, and *tgf-β3*) in wild *M. albus*, respectively.

The animal intestine contains a large number of complex microorganisms, which form a complex symbiotic relationship with the intestine, constituting a microbiological barrier, and changes in their levels are critical in maintaining homeostasis in the intestinal tract. In the present study, the results of alpha and beta diversity analyses showed significant differences in community richness, diversity, and flora structure of the gut microbiota between wild and cultured *M. albus*. Similar results have been reported in wild and cultured fine flounder *Paralichthys adspersus* (10), yellowtail kingfish *Seriola lalandi* (11), kaluga sturgeon *Huso dauricus* (12), Nile tilapia *Oreochromis niloticus* (13), and red cusk-eel *Genypterus chilensis* (14). Studies have shown that the intestinal flora alpha and beta diversity of fish is closely related to their living environment, variation in food habits, and nutritional intake (31, 32). Cao et al. (33) concluded by meta-analysis that, compared with the technical factors, host-associated and environmental factors influenced alpha and beta diversity to a larger extent, and the environmental factors led by diet impacted the alpha and beta diversity of gut bacteria among the host-associated and environmental factors.

In this experiment, at the phylum level, *Proteobacteria*, *Firmicutes*, *Bacteroidetes*, *Actinobacteria*, and *Fusobacteria* were found to be present in the wild and cultured groups, in line with the research on the types of gut microbial composition of healthy *M. albus* (34). The analysis revealed that the abundance of *Firmicutes*, *Bacteroidetes*, *Actinobacteria*, *Cyanobacteria*, and *Verrucomicrobia* in the wild group was significantly higher, and *Chlamydiae* and *Spirochaetes* were lower than those of cultured ones. Many members of *Firmicutes* are beneficial bacteria that produce acetate, butyrate, lactate, and antimicrobial substances that prevent pathogens from interfering with health and help maintain the integrity of the intestinal wall (35). The bacterial community under the classification of *Bacteroidetes* is closely associated with the conversion of organic matter such as proteins and lipids (14), and the increased abundance suggests a greater capacity for energy harvesting by their hosts, which may be due to the wild *M. albus* consuming less and irregular amounts of food in the wild, and they must digest and utilize their food more efficiently to maximize energy, a natural adaptation to their wild condition. *Actinobacteria* that were abundant in hindgut are widely known to produce antimicrobial secondary metabolites within the fish gut that protect the host fish from pathogens *in vivo* (36). Notwithstanding, Piazzon et al. (37) reported decreased gut *Firmicutes* and *Bacteroidetes* and promoted *Proteobacteria* compared with healthy fish after enteritis infection. Furthermore, the abundance analysis showed a shift in the level of *Actinobacteria* in healthy fish to *Proteobacteria* in infected ones (38). At present, the significantly lower abundance of *Firmicutes*, *Bacteroidetes*, and *Actinobacteria* was displayed in the cultured group, although the percentage of *Proteobacteria* in wild and cultured *M. albus* was not significantly different. Therefore, from the point of gut microorganisms, it was speculated that the gut health of wild *M. albus* was superior to that of cultured *M. albus.*

*Cyanobacteria* are mainly distributed in freshwater with a high content of organic matter. The wild environment has more organic matter, which may be one of the main reasons for the higher abundance of *Cyanobacteria* in the wild *M. albus* gut. *Verrucomicrobia* can break down polysaccharides such as mucopolysaccharides and cellulose, which provide energy and nutrients, and also produce short-chain fatty acids such as propionic acid and butyric acid, which are important for intestinal health and immune system regulation (39). The flora under *Spirochaetes* and *Chlamydiae* are mostly pathogenic bacteria that can cause a variety of fish diseases including enteritis and gill mucosal adhesion necrosis, which seriously jeopardize the health of cultured animals (40). Studies have shown that stress can lead to an increase in the abundance of *Spirochaetes* and *Chlamydiae* in the fish gut (41); therefore, it is hypothesized that the ammonia and nitrogen levels in aquaculture water may be higher than those in the wild environment, which, in turn, affects the harmful flora abundance.

At the genus level, higher abundances of *Lactococcus* and *Cetobacterium* and lower abundances of *Pseudomonadaceae_Pseudomonas* and *Spironema* were observed in the wild group than in the cultured group. *Cetobacterium* is commonly found in the intestinal tract of many fish species, and studies have shown a positive correlation with the content of digestive enzymes such as protease and lipase in carnivorous fish (42). *Lactococcus* maintains intestinal acid–base balance by producing lactic acid, which helps to improve digestion and absorption, promoting intestinal health, while as a probiotic it can balance the intestinal flora (43). Mougin and Joyce (44) reported that *Pseudomonadaceae_Pseudomonas* and *Spironema* are associated with intestinal barrier dysfunction and inflammatory bowel disease infections in fish. High abundance means that *M. albus* in cultured environments are more susceptible to infections than those in the wild, possibly due to excessive organic matter in the aquaculture environment, leading to susceptibility to pollution, which allows conditionally pathogenic bacteria to grow and multiply and produce toxins and other disease-causing factors (45). The environmental stresses experienced by fish reared at a high density in culture tanks differ from those experienced by fish living in natural water, with the former being subjected to significantly more stressful stimuli than the latter. It is the frequent exposure of cultured fish to a variety of stressors that tends to lead to a decline in the intestinal health and immunity of cultured fish, increasing their susceptibility to pathogens, and thus manifesting as an increase in the abundance of pathogenic bacteria (46). In addition, the large amount of feed consumed by cultured fish may place a greater burden for the fish gut, which may also contribute to the fact that the intestinal inflammation and intestinal barrier function of farmed M. *albus* are also worse than those of wild ones.

## 5 Conclusion

Overall, the present research showed significant differences in intestinal health between wild and cultured *M. albus* and the main manifestations that wild *M. albus* had higher intestinal digestion, antioxidant capacity, and intestinal barrier functions, including physical, chemical, immunological, and microbiological barriers, than cultured *M. albus*, providing theoretical references for nutrient regulation of the intestinal health of cultured *M. albus*.

## Data availability statement

The original contributions presented in the study are included in the article/supplementary material. Further inquiries can be directed to the corresponding authors.

## Ethics statement

The Animal Ethics Committee of Shanghai Academy of Agricultural Sciences approved all animal procedures. The study was conducted in accordance with the local legislation and institutional requirements.

## Author contributions

HY: Formal analysis, Investigation, Methodology, Writing – original draft. QY: Conceptualization, Data curation, Writing – review & editing. MR: Conceptualization, Data curation, Writing – review & editing. WL: Investigation, Methodology, Writing – review & editing. WH: Resources, Software, Validation, Writing – review & editing. WWH: Visualization, Writing – review & editing. WZ: Funding acquisition, Project administration, Supervision, Writing – review & editing.
